# Foliar Application of Methyl Jasmonate and Chitosan Improve Growth, Yield, and Quality of Guar (*Cyamopsis tetragonoloba* L.) Under Water-Deficit Stress

**DOI:** 10.3390/plants13213099

**Published:** 2024-11-03

**Authors:** Sara Khurizadeh, Ruhollah Naderi, Heidar Meftahizadeh, Saeid Hazrati, Silvana Nicola

**Affiliations:** 1 Department of Plant Production and Genetics, Shiraz University, Shiraz 71964-84334, Iran; khurizadehsara72@gmail.com; 2 Department of Horticultural Sciences, Faculty of Agriculture & Natural Resources, Ardakan University, Ardakan P.O. Box 184, Iran; hmeftahizade@ardakan.ac.ir; 3 Department of Agronomy and Plant Breeding, Faculty of Agriculture, Azarbaijan Shahid Madani University, Tabriz 53714-161, Iran; 4 Department of Agricultural, Forest, and Food Sciences—DISAFA, Horticultural Sciences—INHORTOSANITAS, Vegetable Crops and Medicinal and Aromatic Plants—VEGMAP, University of Torino, 10095 Grugliasco, Italy; silvana.nicola@unito.it

**Keywords:** elicitors, guar, gum, viscosity, water availability

## Abstract

Guar (*Cyamopsis tetragonoloba* L.), a summer legume, is becoming increasingly important as an industrial crop due to its high gum and viscosity content. This study investigated the effects of methyl jasmonate (MeJA), chitosan (CH), and their combination on the growth, yield, and quality of guar under irrigation regimes. A greenhouse experiment was conducted using a factorial design to evaluate the effect of foliar spraying with MeJA (5, 25, and 50 µM), CH (100, 150, and 200 mg/L), their combination (25 µM MeJA + 150 mg/L CH), and control on two commercial guar varieties (RGC-986 and BR-2017) under different irrigation regimes (100%, 70%, and 40% field capacity). The results showed that the exogenous application of MeJA and CH, individually and in combination, significantly enhanced various morphological traits and yield components in guar, including plant height, pod characteristics, seed yield, and root development. Additionally, the combination treatments improved seed quality parameters, such as gum percentage and viscosity content. Leaf analysis revealed increased levels of total phenolic content, total flavonoid, and anthocyanin contents. The BR-2017 variety showed superior performance in most morphological and qualitative traits, demonstrating greater resistance to irrigation regimes. It maintained yield and quality characteristics under water-deficit conditions, particularly when treated with 25 µM MeJA and 150 mg/L CH. The highest gum percentage (33.67%) and viscosity (4768.5 cP) were observed in the RGC-986 variety, along with enhanced levels of secondary metabolites. This study provides new insights into how MeJA, CH, and their combination can improve the yield and quality of guar under water deficit stress conditions. The results suggest that the use of these elicitors, especially in combination, represents an innovative strategy for improving guar production and quality, with potential variety-specific responses to water-deficit stress.

## 1. Introduction

Guar [*Cyamopsis tetragonoloba* (L.) Taub.], a member of the Leguminosae family, is a warm-season annual crop primarily cultivated in India (the largest exporter), Pakistan, Australia, and several Southern European countries, with the USA being the biggest importer [1]. Guar is known for its fast growth, bushy structure, and deep root system, making it suitable for human food, animal feed, green manure, and vegetables. It thrives in sandy loam soils and arid to semi-arid regions, demonstrating resilience to drought conditions [2,3,4]. The endosperm of guar seeds contains approximately 37% guar gum, a polysaccharide specifically classified as a polygalactomannan. This substance is soluble in water and can significantly increase the viscosity of aqueous solutions, even at low concentrations (≤1% *w*/*v*) [5,6,7]. Guar gum products offer multiple health benefits, including blood sugar reduction, weight management, and potential anti-cancer effects. Additionally, they exhibit significant antioxidant activity, helping to prevent oxidative damage and reduce the risk of various diseases [8]. The industrial significance of guar has risen due to the polysaccharides found in its seeds, particularly in the production of food stabilizers, papermaking, textile printing, mining explosives, drilling, and various sectors such as pharmaceuticals, cosmetics, and oil and gas exploration [9,10,11]. The high content of galactomannan in the guar endosperm enhances its utility in these applications, as its ability to form hydrogen bonds with water molecules significantly contributes to its effectiveness. Notably, guar gum has exceptional water-thickening capacity and is eight times greater than that of other agents, such as cornstarch or gums like gum arabic and tragacanth. This efficiency allows for the use of smaller amounts of guar gum to achieve the desired viscosity, resulting in lower costs and making it a more economical option. Global demand for guar gum has risen dramatically, driven primarily by its essential role in hydraulic fracturing for shale gas extraction. This unprecedented demand has led to a steep price increase of around 230%. As a result, India, which dominates global guar production, has increased its exports by 75% to meet this growing market demand [10,11,12]. Global climate change and adverse environmental conditions are severely limiting agricultural productivity, with drought emerging as one of the most devastating and unpredictable stressors. Rising temperatures and increasingly severe droughts have made the cultivation of drought-tolerant crops increasingly important for the sustainability of agriculture. Among these resilient crops, guar is particularly promising. Water scarcity, a key challenge of climate change, can reduce global crop yields by up to 50% and is responsible for almost half of all crop losses worldwide [13,14]. In situations where water is limited in the environment, guar can be a very suitable option as a plant with the ability to tolerate water shortages [15]. Efficient water management is vital for the sustainable cultivation of guar. While adequate water is essential, excessive irrigation can negatively affect seed yield in indeterminate crops like guar. Over-irrigation may lead to excessive vegetative growth and delayed seed formation. Water-deficit stress is generally detrimental to guar yield, and global guar genetic resources have not been adequately characterized for drought tolerance. Therefore, the identification of drought tolerant genotypes has become particularly important for sustainable guar production. Several studies have documented the extensive effects of water-deficit stress on guar plant development and yield components. Research has consistently shown that water deficit significantly affects the vegetative growth of guar, particularly reducing plant height and root development [16]. In addition to morphological effects, water-deficit stress reduces reproductive success by reducing pod formation and seed yield [17]. A comprehensive study showed that water-deficit conditions not only reduced physical growth parameters but also affected the commercial value of guar by reducing gum yield and viscosity [18]. Furthermore, water-deficit stress negatively affects the nutritional and industrial properties of the plant, resulting in reduced galactomannan content, protein content, and seed weight [19]. Taken together, these findings underscore how water availability is a critical determinant of both the agronomic performance and economic value of guar crops.

Optimal guar seed production typically requires around 900 mm of rainfall [20,21,22,23]. It is important to note that excess water can potentially reduce maximum yield [21]. Therefore, careful irrigation practices are necessary to balance plant water requirements with optimal seed production. Recent studies have demonstrated guar adaptability to water-deficit stress [24,25,26], and ongoing research is exploring drought mitigation strategies using natural substances such as methyl jasmonate (MeJA) hormones [27] and chitosan (CH) biostimulants [28] to enhance resilience to abiotic stress, particularly in water-scarce regions.

MeJA is a hormonal compound that stimulates plant development and defense mechanisms by influencing endogenous hormone levels and various signaling pathways in response to biotic and abiotic stresses [27,28,29]. It counteracts osmotic stress by regulating the uptake of minerals and organic compounds, suppressing the absorption of toxic ions, and reducing oxidative stress through the activation of antioxidant systems [30]. When applied exogenously at optimal concentrations, MeJA can effectively mitigate damage to plants caused by drought, salinity, and extreme temperatures. Foliar application of MeJA significantly improves recovery from drought stress through multiple physiological and biochemical pathways. MeJA promotes drought resistance by increasing relative water content, stimulating proline accumulation, and enhancing antioxidant defenses. This versatile plant growth regulator modulates stress responses by upregulating both organic osmolytes and oxidative enzyme activities. In particular, MeJA activates the phenylpropanoid pathway, leading to increased synthesis of non-enzymatic antioxidants, including anthocyanins and phenolic compounds, which further enhance plant defense against water-deficit conditions [31,32,33]. CH, a natural biopolymer derived from chitin, is the second most abundant polysaccharide on Earth, following cellulose [34]. As a non-toxic, biodegradable, and environmentally friendly material, it has gained traction as a growth promoter in agriculture [27]. CH possesses light-reflecting and antiperspirant properties that help reduce water loss in plants while facilitating cooling [35]. Research indicates that low concentrations of CH can alleviate the effects of water-deficit stress, enhance plant growth and yield, and provide protection against oxidative stress. CH and jasmonic acid synergistically activate genes encoding key defense proteins, including phenylalanine ammonia lyase (PAL) and protease inhibitors. CH exposure triggers a cascade of phytohormone responses, stimulating the biosynthesis of both jasmonic acid and abscisic acid. Through the octadecanoid pathway, CH treatment enhances the accumulation of jasmonic acid and its precursor, 12-oxo phytodienoic acid. This hormonal cascade culminates in abscisic acid-mediated stomatal closure, a process regulated by hydrogen peroxide-dependent signaling pathways [35]. Guar, a leguminous crop capable of symbiotic nitrogen fixation, thrives in less fertile and marginal soils with minimal agricultural inputs. This trait not only enhances soil fertility in an economical and sustainable manner but also benefits subsequent crop yields. As such, guar presents a valuable option for cultivation in Iran’s semi-arid regions, characterized by poor soil quality and water scarcity [36,37]. However, despite guar adaptability, the specific threshold at which water-deficit stress significantly impacts its yield remains an area requiring further research.

Despite the industrial importance of guar, there is a notable gap in the literature concerning the role of the elicitors on guar growth under different irrigation regimes. Specifically, the simultaneous application of MeJA and CH to guar plants under water-deficit stress has not been adequately explored. This gap presents an opportunity for a more comprehensive investigation. The primary objective of this study is to examine the effects of simultaneous exogenous foliar application of MeJA, CH, and their combination (MeJA + CH) on the resilience of guar to water-deficit stress. Moreover, an innovative and underexplored approach will be evaluated. The research will specifically focus on morphological responses, yield components, and viscosity of guar under these conditions. The findings will offer valuable insights for researchers, growers, and policymakers aiming to enhance guar production in water-limited environments and potentially expand its cultivation in semi-arid regions.

## 2. Materials and Methods

Seeds of guar varieties RGC-986 and BR-2017 were obtained from the Agricultural Research Centre in Rajasthan State, India. Methyl jasmonate (MeJA), specifically 3-oxo-2-(2-pentenyl) cyclopentaneacetic acid, methyl ester (Merck Company, Darmstadt, Germany, 95%), and chitosan (CH), in the form of deacetylated chitin, poly(-glucosanine) (Merck Company, Darmstadt, Germany, medium molecular weight), were evaluated for their effectiveness in mitigating the effects of different irrigation regimes. Tween 80 (Merck Company, Darmstadt, Germany) served as a wetting agent in all treatments at a concentration of 0.1% (*v*/*v*).

### 2.1. Experimental Design and Treatments

This study was conducted in 2023 at the research greenhouse of the Department of Plant Production and Genetics, School of Agriculture, Shiraz University, Fars Province, Iran (46°52′ E, 29°7′ N, altitude 1810 m). A factorial experiment was carried out using a completely randomized design with three replications. The effects of irrigation regimes, MeJA, CH, and their combined application on two commercial guar varieties, RGC-986 (V1) and BR-2017 (V2), were investigated. Irrigation regimes were categorized into three levels for each guar variety: I_1_-well watered (control) (100% field capacity, (FC), I_2_-70% FC (moderate water-deficit stress), and I_3_-40% FC (severe water-deficit stress), determined using the pot weighing method. Within each guar variety, treatments were further stratified into twenty-four sub-groups: (a) MeJA solutions prepared according to [27] at concentrations of 5 µM (MJ1), 25 µM (MJ2), and 50 µM (MJ3); (b) CH solutions prepared as per [34] at concentrations of 100 mg/L (CH1), 150 mg/L (CH2), and 200 mg/L (CH3), using 1% acetic acid for preparation; (c) a combined treatment of 25 µM MeJA and 150 mg/L CH (MJ2 + CH2); and (d) a non-treated control group. This arrangement resulted in eight distinct treatment combinations, each replicated three times, thus having 144 pots in total.

Five guar seeds were planted in each pot. After germination and establishment, the plants in each pot were thinned, reducing the number to three. The pots were subjected to water-deficit stress starting at the four-leaf stage, with irrigation applied according to crop capacity. Initially, the pots were watered to 100% FC. In the following days, all pots were weighed, and water was added based on the desired FC, considering variations in pot weight and the amount of drained water. Foliar spraying treatments were applied at three critical developmental stages during the 2023 growing season: vegetative stage (four-leaf stage, 53 days after sowing), flowering stage (80% full flowering, 85 days after sowing), and pod formation stage (110 days after sowing). Fresh solutions of MeJA and CH (in 1% acetic acid) in distilled water were prepared at their respective indicated concentrations before each application.

To minimize cross-contamination, treated pots were temporarily moved to an isolated section of the greenhouse for application. The solutions were applied using a hand sprayer to the point of run-off at 08:00 a.m. After a 30 min post-treatment period, the pots were returned to their original positions. This procedure was repeated for all treatments and concentrations within the same day. Control plants received only a foliar application of distilled water.

### 2.2. Plant Growth Conditions

The trials were conducted in an automated glass greenhouse equipped with integrated heating, ventilation, and seasonal shading systems to maintain optimal growing conditions for the guar plants. Environmental parameters were precisely controlled, with daytime temperatures maintained at 24 °C and nighttime temperatures at 16 °C. The photoperiod was regulated to 16/8 h (light/dark), while relative humidity was stabilized at 60 ± 5%. Supplemental artificial lighting was provided in conjunction with natural daylight to ensure consistent light exposure throughout the growing season, with the duration adjusted to meet the physiological needs of the plants. Seeds were sown into plastic pots measuring 15 cm in diameter and 30 cm in height, each filled with 5 kg of soil. The soil was a loamy sand with the following characteristics: pH of 7.68, electrical conductivity (EC) of 0.75 dS m^−1^, total nitrogen (N) content of 0.21%, available phosphorus (P) of 17 mg kg^−1^, available potassium (K) of 571 mg kg^−1^, and 0.65% organic matter.

To determine the field capacity (FC) of the experimental soil, a pot was filled with oven-dried soil (at 105 °C for 24 h), and then the pot was saturated with water until drainage occurred at the bottom. Then, the pot was covered with a plastic sheet to prevent evaporation. The pot allowed draining the excess water for 72 h. Thus, the water content of the soil has reached its FC point, and it can be measured by dividing the weight of this wet soil by its initial oven-dried weight [4]. There were three irrigation treatments, namely, 100, 70, and 40% of FC. Each pot was weighed every 3 days. Then, irrigation was carried out by adding an appropriate amount of water using a beaker so that the soil reached the field capacity point for 100% FC treatments. For 70% and 40% FC treatments, the amount of water added to the pots was that much to increase the soil water content to 70 and 40% FC, respectively.

Seeds were sown on February 2. To control aphid populations, the insecticide imidacloprid^®^ was applied as needed throughout the study.

### 2.3. Data Collection and Measurements

#### 2.3.1. Morphological Measurements

Plants were harvested 150 days after sowing, at physiological maturity (when the pods turned yellow). From each treatment replication, three plants were randomly selected. Measurements were taken for each plant individually, and means were calculated for each pot. Plant height was measured from the soil surface to the top of the plant using a ruler. For biomass, plants were oven-dried at 72 °C for 48 h before weighing. Roots were separated, washed, and measured for length, then surface-dried and oven-dried at 60 °C for 48 h to determine dry weight. The number of pods per plant was counted, and five randomly selected pods were used to measure pod length and the number of seeds per pod. The harvested pods were dried, threshed, and 100 seeds were weighed using a digital balance, with results extrapolated to determine the 1000-seed weight. All length measurements were recorded in centimeters, and a precision balance enabled dry seed weight measurement.

#### 2.3.2. Qualitative Characteristics

##### Determination of Total Phenolic Content

To determine the total phenolic content based on the Folin–Ciocalteu method, 0.1 g of dry leaf samples was homogenized in deionized water (1 mL). A 0.1 mL aliquot of this solution was mixed with 2 mL of 2% sodium carbonate (Na_2_CO_3_), 2.8 mL of deionized water, and 0.1 mL of 50% Folin–Ciocalteu reagent. The mixture was incubated at room temperature for 30 min. Absorbance was measured at 765 nm against a deionized water blank using a spectrophotometer (Bright Technologies Inc., Chandkheda, India). Gallic acid was regarded as the standard, as suggested by Miliauskas et al. [38]. The results were reported as mg of gallic acid equivalent per g of dry weight.

##### Determination of Total Flavonoid Content

The total flavonoid content of guar plants was measured using a spectrophotometer, following the aluminum chloride colorimetric method as described by Chang et al. (2002) [39]. In brief, 0.1 g of leaf samples was dissolved in 1 mL of deionized water. Then, 0.5 mL of this solution was combined with 1.5 mL of 95% alcohol, 0.1 mL of 10% aluminum chloride hexahydrate (AlCl_3_), 0.1 mL of 1 M potassium acetate (CH_3_COOK), and 2.8 mL of deionized water. After incubating the mixture at room temperature for 40 min, the absorbance was measured at 510 nm against a deionized water blank using a spectrophotometer. Quercetin served as the standard for comparison. The results were reported as mg of quercetin equivalent to g dry weight.

##### Determination of Anthocyanin

Anthocyanin measurement was based on the absorbance of filtered extract obtained by exposing 0.2 g of dry leaves to acidic methanol (a solution of methanol and hydrochloric acid in a ratio of 99:1 at a wavelength of 550 nm with a spectrophotometer and using the extinction coefficient (ε = 33,000 mol^−2^ cm^−1^) [40].

##### Determination of Gum Content

Gum separation was conducted following the methods of Meftahizadeh et al. [41], with some modifications. Initially, the seeds were soaked in water for eight hours, after which the seed shells were removed by rubbing the surface. Subsequently, pressure was applied to detach the seed gum from the embryo and endosperm. The extracted gum underwent an eight-hour drying process in an oven set at 50 °C. Finally, the gum was weighed, and its percentage relative to the embryo and endosperm was calculated.

##### Determination of Viscosity

Following the method of Khalid Sabahlkheir et al. [23], the obtained gum powder was dissolved in water for hydration. The mixture was then centrifuged at 5000 rpm for 15 min to remove impurities. The resulting gum solution was combined with ethanol at a ratio of 3:1 and left to sit for 50 min. This step loosened the bond between the gum and water molecules, facilitating separation and causing the formation of white coils in the solution. These white coils were then separated from the water and dried in an oven at 40 ºC for 6 h, resulting in pure guar gum. To evaluate viscosity, solutions of varying concentrations were prepared from the purified gum. Specifically, a 1% concentration was made by mixing 1 g of gum powder with 99 mL of deionized water and stirring magnetically for 24 h to ensure complete hydration. After adjusting the D-V-3 ultra-viscometer (Lamy Rheology, Champagne-sur-Marne, France), the viscosity of the gum at this concentration and others was investigated.

### 2.4. Statistical Analysis

Data analysis was conducted using SAS software version 9.4 (SAS Institute, Cary, NC, USA). Data from three replications were first assessed for normality. Analysis of variance (ANOVA) with a general linear model was employed to evaluate both main and interaction effects. To enhance data interpretation, slicing was performed at a variety level. When the F-test indicated significant differences, the mean values of the main effects were compared using the Tukey HSD (honestly significant difference) test (*p* ≤ 0.01). Significant interactions were further analyzed using the slicing method. In cases of significant three-way interactions, these were prioritized in the discussion. For significant two-way interactions without notable three-way interactions, only the two-way interactions were discussed. When no significant interactions were detected, only the main effects were considered. This hierarchical approach to interaction analysis ensured a comprehensive yet focused interpretation of the results. Graphs illustrating the findings were generated using Excel 2021.

## 3. Results

### 3.1. Analysis of Variance

The ANOVA analysis of all the measured parameters, i.e., growth, yield, and quality of the guar varieties, was affected significantly by the main effects, two-way and three-way interactions between the different levels of elicitors, varieties, and irrigation regimes (Table 1). Interactions and main effects are discussed below in order of statistical significance, from the highest-level interactions to the main effects of treatments. Where there are two- or three-way interactions for each measured characteristic, this means that the interpretation of the main effects is incomplete or avoided.

### 3.2. Plant Height and Pod Length

Water-deficit stress significantly reduced plant height in both guar varieties compared to well-watered conditions (Table 2 and Table 3). However, the highest plant height was recorded in variety BR-2017, and foliar applications of MeJA, CH, and MeJA + CH helped mitigate the stress effects, preventing a sharp reduction in plant height. The maximum height (70.33 cm) was observed in BR-2017 under well-watered conditions with 25 µM MeJA, representing a 39.32% increase compared to the control (42.67 cm) (Table 3).

Similarly, pod length was largest in BR-2017 and decreased under stress in both varieties (Table 2 and Table 3). Spraying with MeJA, CH, and MeJA + CH alleviated the stress effects, reducing the severity of pod length reduction. The maximum pod length (7.17 cm) was achieved in BR-2017 under well-watered conditions combined with 200 mg/L CH, showing a 32.86% increase compared to the control (4.67 cm) (Table 3).

### 3.3. Pod Number, Number of Seeds Pod^−1^ and Seed Yield Per Plant

Variety BR-2017 produced the highest number of pods, but water-deficit stress had a significant negative effect on this trait in two varieties (Table 4 and Table 5). However, foliar spraying with MeJA, CH, or MeJA + CH increased the pod count. The maximum pod number (5.33) was observed in BR-2017 under well-watered conditions with foliar application of 50 µM MeJA, showing a 37.52% increase compared to the control (3.33) (Table 5). Similarly, BR-2017 produced the highest number of seeds pod^−1^, though this also decreased under stress. The maximum number of seeds pod^−1^ (5.33) was recorded under well-watered conditions with 50 µM MeJA, representing a 43.71% increase compared to the control (3) (Table 5). Seed yield per plant was also highest in BR-2017 but decreased under stress in both varieties (Table 4 and Table 5). However, foliar spraying with MeJA, CH, or MeJA + CH mitigated the stress effects and prevented a severe reduction in seed yield. The highest seed yield per plant (0.97 g) in BR-2017 was achieved under well-watered conditions with 25 µM MeJA, showing a 62.88% increase compared to the control (0.36 g) (Table 5).

### 3.4. 1000-Seed Weight and Biomass

**Figure 1:**
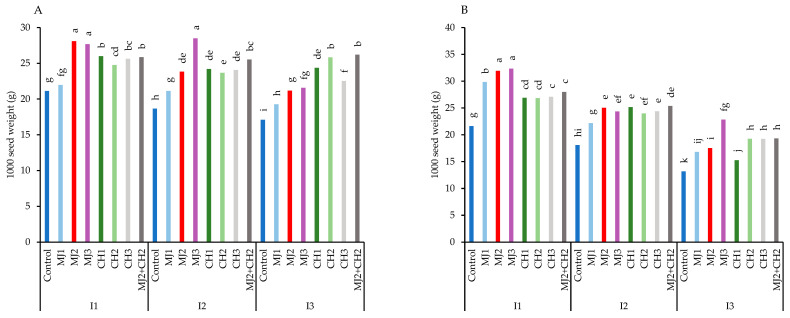
1000-seed weight of guar varieties ((**A**): RGC-986 and (**B**): BR-2017) as affected by elicitors and irrigation regimes. I1, I2, and I3 represent irrigation regimes as 100%, 70%, and 40% FC, respectively. MJ1, MJ2, MJ3 represent 5, 25, 50 µM of methyl jasmonate. CH1, CH2, CH3 represent 100, 150, and 200 mg/L chitosan. MJ2+ CH2 represents combination of 25 µM MeJA and 150 mg/L CH. Means within the same column with the same letter are not significantly different according to Tukey’s honest significant difference (HSD) test (*p* ≤ 0.05).

The results showed that the highest 1000-seed weight was observed in BR-2017. Although water-deficit stress caused a decline in 1000-seed weight across both varieties, foliar spraying with MeJA, CH, and MeJA + CH helped mitigate the stress effects and prevented a sharp reduction in seed weight (Figure 1A,B). In BR-2017, the highest 1000-seed weight (31.94 g) was achieved with 50 µM MeJA under well-watered conditions, representing a 32.34% increase compared to the control (21.61 g) (Figure 1B).

**Figure 2:**
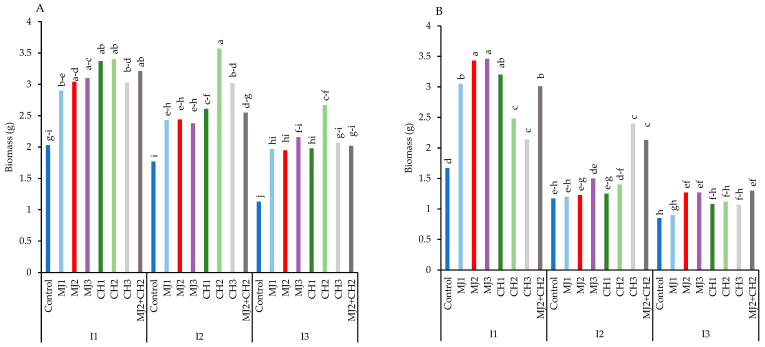
Biomass of guar varieties ((**A**): RGC-986 and (**B**): BR-2017) as affected by elicitors and irrigation regimes. I1, I2, and I3 represent irrigation regimes as 100%, 70%, and 40% FC, respectively. MJ1, MJ2, MJ3 represent 5, 25, 50 µM of methyl jasmonate. CH1, CH2, CH3 represent 100, 150, and 200 mg/L chitosan. MJ2+ CH2 represents combination of 25 µM MeJA and 150 mg/L CH. Means within the same column with the same letter are not significantly different according to Tukey's honest significant difference (HSD) test (*p* ≤ 0.05).

The results indicated that the highest biomass was associated with variety RGC-986, although it declined under water-deficit stress in both varieties (Figure 2A,B). However, foliar spraying with MeJA, CH, and MeJA + CH mitigated water-deficit stress and ameliorated the decline in biomass. The maximum biomass (3.57 g) was recorded in RGC-986 under moderate water-deficit stress, with 150 mg/L CH, representing a 43.13% increase than the control (2.03 g) (Figure 2A).

### 3.5. Root Length and Root Dry Weight

**Figure 3:**
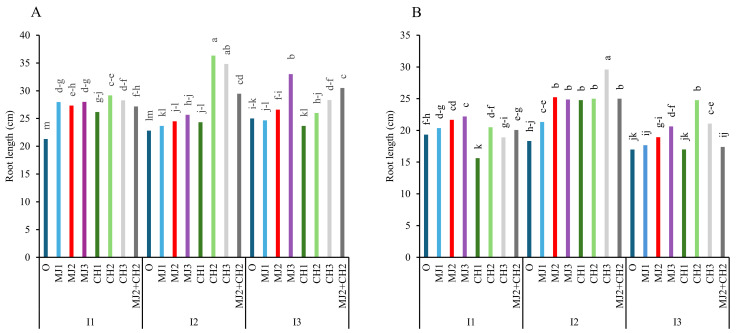
Root length of guar varieties ((**A**): RGC-986 and (**B**): BR-2017) as affected by elicitors and irrigation regimes. I1, I2, and I3 represent irrigation regimes as 100%, 70%, and 40% FC, respectively. MJ1, MJ2, MJ3 represent 5, 25, 50 µM of methyl jasmonate. CH1, CH2, CH3 represent 100, 150, and 200 mg/L chitosan. MJ2+ CH2 represents combination of 25 µM MeJA and 150 mg/L CH. Means within the same column with the same letter are not significantly different according to Tukey's honest significant difference (HSD) test (*p* ≤ 0.05).

Although the application of MeJA, CH, and MeJA + CH reduced the impact of water-deficit stress and helped prevent a sharp decline in root length in both varieties (Figure 3A,B), the results showed that variety RGC-986 had the longest roots, which shortened under stress in both varieties. Under moderate stress, RGC-986 treated with 150 mg/L CH achieved the maximum root length (36.33 cm), representing a 41.28% increase compared to the control (21.33 cm) (Figure 3A).

**Figure 4:**
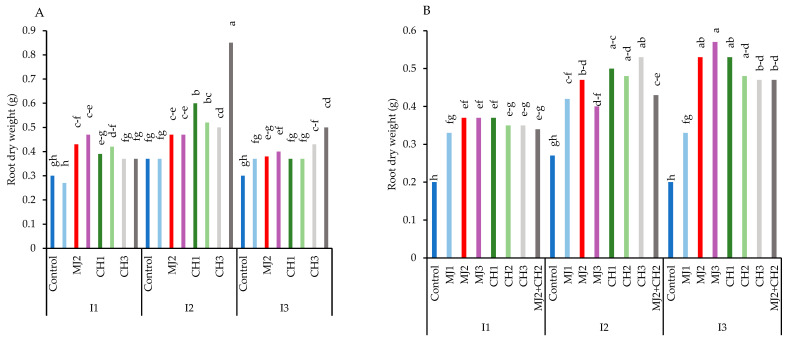
Root dry weight of guar varieties ((**A**): RGC-986 and (**B**): BR-2017) as affected by elicitors and irrigation regimes. I1, I2, and I3 represent irrigation regimes as 100%, 70%, and 40% FC, respectively. MJ1, MJ2, MJ3 represent 5, 25, 50 µM of methyl jasmonate. CH1, CH2, CH3 represent 100, 150, and 200 mg/L chitosan. MJ2+ CH2 represents combination of 25 µM MeJA and 150 mg/L CH. Means within the same column with the same letter are not significantly different according to Tukey's honest significant difference (HSD) test (*p* ≤ 0.05).

The results showed that RGC-986 had the highest root dry weight. The root dry weight decreased under water-deficit stress in both varieties (Figure 4A,B). However, the application of MeJA, CH, and MeJA + CH helped mitigate the effects of stress and prevented a significant decline in root dry weight. Under moderate stress conditions, when treated with 25 µM MeJA + 150 mg/L CH, variety RGC-986 exhibited the largest root dry weight (0.85 g), representing a 64.70% increase compared to the control (0.30 g) (Figure 4A).

### 3.6. Total Phenolic Content, Flavonoid, and Anthocyanin Content

**Figure 5:**
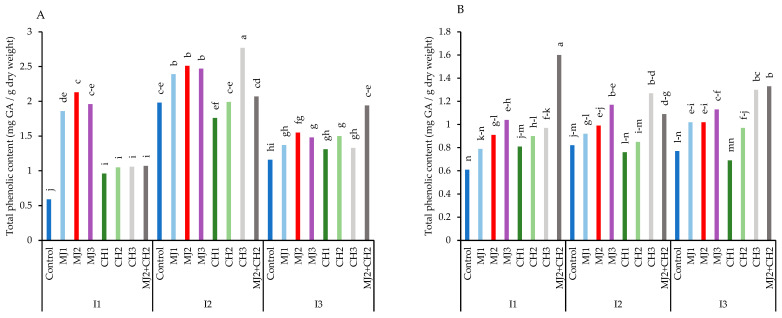
Total phenolic content of guar varieties ((**A**): RGC-986 and (**B**): BR-2017) as affected by elicitors and irrigation regimes. I1, I2, and I3 represent irrigation regimes as 100%, 70%, and 40% FC, respectively. MJ1, MJ2, MJ3 represent 5, 25, 50 µM of methyl jasmonate. CH1, CH2, CH3 represent 100, 150, and 200 mg/L chitosan. MJ2+ CH2 represents combination of 25 µM MeJA and 150 mg/L CH. Means within the same column with the same letter are not significantly different according to Tukey's honest significant difference (HSD) test (*p* ≤ 0.05).

When compared two varieties, water-deficit stress considerably increased the total phenolic content (Figure 5A,B). Accordingly, moderate deficit stress along with 150 mg/L CH caused the variety RGC-986 to obtain maximum total phenolic content (2.77 mg GA/g dry weight), which increased by 78.70% compared to the control (0.59 mg GA/g dry weight). The results also showed that spraying MeJA, CH, and MeJA + CH increased the total phenolic content in water-deficit situations (Figure 5A).

**Figure 6:**
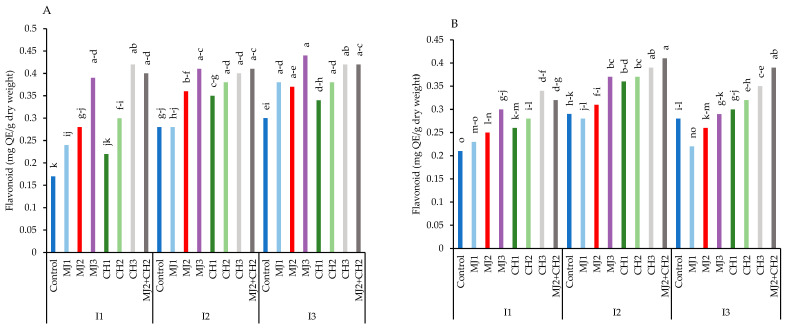
Total flavonoid of guar varieties ((**A**): RGC-986 and (**B**): BR-2017) as affected by elicitors and irrigation regimes. I1, I2, and I3 represent irrigation regimes as 100%, 70%, and 40% FC, respectively. MJ1, MJ2, MJ3 represent 5, 25, 50 µM of methyl jasmonate. CH1, CH2, CH3 represent 100, 150, and 200 mg/L chitosan. MJ2+ CH2 represents combination of 25 µM MeJA and 150 mg/L CH. Means within the same column with the same letter are not significantly different according to Tukey's honest significant difference (HSD) test (*p* ≤ 0.05).

While MeJA, CH, and MeJA + CH spraying mitigated the effects of stress and prevented a notable increase in flavonoids, variety RGC-986 showed the highest level of flavonoid, which increased under stress in both varieties (Figure 6A,B). Thus, variety RGC-986 achieved maximum flavonoid content (0.44 mg QE/g dry weight) under moderate stress, when treated with 25 MeJA + 150 mg/L of CH, thus being 61.36% greater than the control (0.17 mg QE/g dry weight) (Figure 6A).

**Figure 7:**
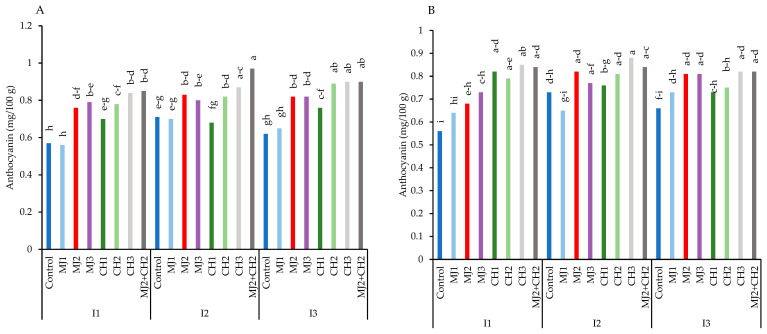
Anthocyanin of guar varieties ((**A**): RGC-986 and (**B**): BR-2017) as affected by elicitors and irrigation regimes. I1, I2, and I3 represent irrigation regimes as 100%, 70%, and 40% FC, respectively. MJ1, MJ2, MJ3 represent 5, 25, 50 µM of methyl jasmonate. CH1, CH2, CH3 represent 100, 150, and 200 mg/L chitosan. MJ2+ CH2 represents combination of 25 µM MeJA and 150 mg/L CH. Means within the same column with the same letter are not significantly different according to Tukey's honest significant difference (HSD) test (*p* ≤ 0.05).

Also, severe water-deficit stress significantly reduced the anthocyanin content, but moderate stress increased compared to well-watered conditions in both varieties (Figure 7A,B). The results showed the highest amount of anthocyanin content in variety RGC-986, which resulted from spraying MeJA + CH. Maximum anthocyanin content (0.97 mg/100 g) was achieved in variety RGC-986 under moderate stress, when treated with 25 µM + 150 mg/L CH, thus showing an increase of 41.23% compared to the control (0.57 mg/100 g) (Figure 7A).

### 3.7. Gum Percentage and Viscosity

**Figure 8:**
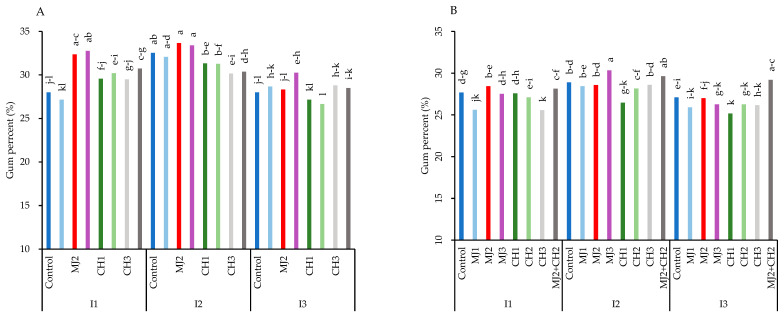
Gum percentage of guar varieties ((**A**): RGC-986 and (**B**): BR-2017) as affected by elicitors and irrigation regimes. I1, I2, and I3 represent irrigation regimes as 100%, 70%, and 40% FC, respectively. MJ1, MJ2, MJ3 represent 5, 25, 50 µM of methyl jasmonate. CH1, CH2, CH3 represent 100, 150, and 200 mg/L chitosan. MJ2+ CH2 represents combination of 25 µM MeJA and 150 mg/L CH. Means within the same column with the same letter are not significantly different according to Tukey’s honest significant difference (HSD) test (*p* ≤ 0.05).

Severe water-deficit stress significantly reduced gum percentage, but moderate stress increased it compared to well-watered conditions in both varieties (Figure 8A,B). The results showed that the highest amount of gum percentage was obtained in variety RGC-986, as spraying MeJA, CH, and MeJA + CH reduced the effects of stress and prevented a sharp decrease in gum percentage. The maximum gum percentage (33.67%) was obtained in variety RGC-986 under moderate stress, when treated with 25 µM MeJA, thus showing an increase of 16.83% compared to the control (28%) (Figure 8A). The MeJA + CH treatment caused variety BR-2017 to have a high gum percentage under moderate and severe stress, although its amount was less than that of variety RGC-986 (Figure 8A).

**Figure 9:**
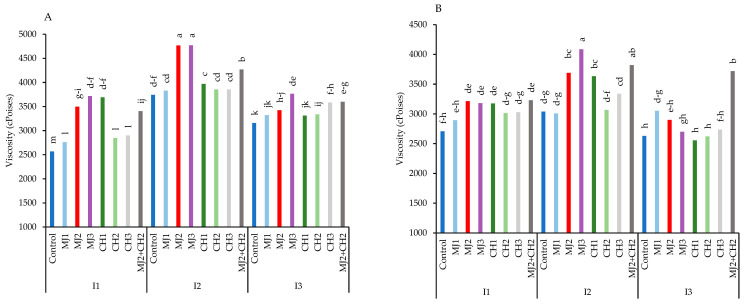
Viscosity of guar varieties ((**A**): RGC-986 and (**B**): BR-2017) as affected by elicitors and irrigation regimes. I1, I2, and I3 represent irrigation regimes as 100%, 70%, and 40% FC, respectively. MJ1, MJ2, MJ3 represent 5, 25, 50 µM of methyl jasmonate. CH1, CH2, CH3 represent 100, 150, and 200 mg/L chitosan. MJ2+ CH2 represents combination of 25 µM MeJA and 150 mg/L CH. Means within the same column with the same letter are not significantly different according to Tukey's honest significant difference (HSD) test (*p* ≤ 0.05).

The results indicated that the variety RGC-986 exhibited the highest viscosity, which increased under moderate stress but decreased under severe stress in both varieties. However, the application of MeJA, CH, and MeJA + CH helped mitigate the effects of stress and prevented a sharp decline in viscosity in both varieties (Figure 9A,B). The maximum viscosity (4770 cPoises) was observed in RGC-986 under moderate stress with 50 µM MeJA, representing a 346.14% increase compared to the control (2569 cPoises) (Figure 9A). The second most effective treatment was the combined application of MeJA + CH under moderate stress, resulting in significant increases in viscosity, i.e., 66.15% in RGC-986 (4268.5 cPoises) and 29.08% in BR-2017 (3818.5 cPoises), compared to their respective controls (Figure 9A).

## 4. Discussion

### 4.1. Increased Guar Growth and Seed Yield

Water-deficit stress significantly impacts plant physiology and growth, leading to reduced root water uptake, increased dehydration, and limited nutrient absorption, which adversely affect various growth characteristics [41,42,43]. As water stress intensifies, leaf dimensions decrease, resulting in a diminished photosynthetic capacity [43]. This stress is typically associated with reduced plant height, lower dry matter production, decreased photosynthesis, diminished nutrient storage in stems and vegetative organs, and ultimately reduced plant yield [44].

Our study demonstrated that treatments with MeJA, CH, and their combination (MeJA + CH) positively influenced the morphophysiological and yield traits of guar plants, particularly under MeJA treatment. These improvements can be attributed to several mechanisms, including the transcriptional activation of genes encoding phenylalanine ammonia lyase and protease inhibitors; the induction of abscisic acid and jasmonic acid synthesis by CH, which enhances stomatal control and water uptake regulation; and the activation of multiple defense responses, such as kinase gene activation and oxidative burst [45,46,47,48,49]. The beneficial effects of CH and MeJA observed in this study are consistent with findings in various crops. Similar improvements in growth, yield, quality, and drought resistance have been documented in other important agricultural species, including soybean (*Glycine max*) [35], maize (*Zea mays*) [31], and white lupin (*Lupinus termis* L.) [42]. These consistent results across multiple legume and cereal species underscore the broad applicability of these biostimulants in enhancing crop resilience. Furthermore, MeJA plays a crucial role in increasing the relative water content in plant shoots and altering biochemical and physiological characteristics, thereby enhancing drought tolerance [12].

Collectively, these findings suggest that MeJA, CH, and their combination activate multiple physiological and biochemical pathways that enhance plant resilience to water-deficit stress, offering potential strategies to mitigate the impacts of water scarcity on guar production. Our results confirm these observations, showing that reduced water availability negatively affects several growth parameters in guar varieties, including plant height, pod length, number of pods per plant, number of seeds per pod, biomass, root length, and root dry weight. These findings are consistent with prior research on guar plants under water-deficit conditions [32,33,34].

In particular, our study reveals that these growth characteristics are significantly influenced by varying concentrations of MeJA and CH. A comparison of the two varieties showed that MeJA and CH, acting as elicitors, could mitigate the adverse effects of both moderate and severe water stress, thereby improving guar growth and yield. Even under severe stress conditions (40% FC), different concentrations of these elicitors improved the studied traits and alleviated the effects of water stress on all components and yield parameters. These results are particularly relevant in the context of crop productivity in semi-arid areas, where water stress is a critical limiting factor [45]. They also suggest potential applications of MeJA and CH in enhancing plant resilience and productivity within irrigation regimes. The use of MeJA and CH treatments shows strong commercial viability, with initial investment in application infrastructure offset by significant improvements in guar yield and quality. Their natural origin, proven efficacy, and increasing availability of low-cost formulations make them practical alternatives for sustainable agriculture, particularly for crops under water-stressed conditions.

Additionally, our results indicated that the foliar application of MeJA or CH under both water-deficit and well-watered conditions promoted plant growth and development, leading to improved morphological characteristics and reduced biomass loss due to water-deficit stress. Therefore, these treatments could be employed in water-restricted areas to sustain guar production. These results align with previous studies demonstrating that foliar applications of CH [34] and MeJA [27] enhanced vegetative growth and yield in other legume crops. This improvement may be attributed to increased stomatal conductance, photosynthetic rates, and antioxidant enzyme activity associated with these treatments [46,47,48].

Our findings on the mitigating effects of MeJA and CH on water stress in guar are consistent with and extend previous research on various legumes and cereals. For example, in *Glycine max*, MeJA (20 µM) improved plant height under water-deficit stress (80% FC) [12], while in *Phaseolus vulgaris* L., CH (100–200 mg/L) enhanced growth parameters under similar conditions (40% FC) [49]. Similarly, CH application (50 mg/L) increased pod and seed numbers in *Lupinus termis* L. under drought stress [42] and improved plant height in *Triticum aestivum* across different irrigation regimes [50].

Both CH and MeJA, individually and in combination, improved growth parameters in two guar varieties under severe water-deficit stress, with MeJA concentrations of 25 and 50 µM being most effective in increasing pod numbers in the RGC-986 and BR-2017 varieties, respectively. These results indicate that foliar application of MeJA or CH under both water-deficit and well-watered conditions promotes plant growth and development, enhances morphological characteristics, and reduces drought-induced biomass loss. The consistency of our results with studies on other crops underscores the potential of these treatments as a strategy to sustain guar production in water-stressed areas and presents a promising approach to improving crop resilience in challenging environmental conditions.

### 4.2. Increased Secondary Metabolites, Gum Percentage, and Viscosity Resulting in High Quality

The enhancement of secondary metabolites, such as total phenolic content, flavonoid, and anthocyanin, observed in our study suggests a promising approach to improving guar quality, particularly in terms of gum content and viscosity. CH plays a vital role in this process by activating stress transduction systems through secondary messengers, which improves physiological responses and mitigates the negative impacts of abiotic stressors [29]. Specifically, CH application leads to increased photosynthetic rates, promotes stomatal closure through ABA synthesis, enhances antioxidant enzyme systems via nitric oxide and hydrogen peroxide signaling pathways, and induces the production of organic acids, sugars, amino acids, and other metabolites crucial for energy metabolism, osmotic regulation, and stress signaling. Additionally, when applied topically, CH acts as an antitranspirant, reducing water consumption and providing protection against other adverse effects [29].

Similarly, MeJA, a signaling hormone, aids in stress reduction [47] by alleviating oxidative stress through the activation of antioxidant mechanisms that scavenge reactive oxygen species (ROS). The observed synergistic effect of MeJA and CH on guar plants under water stress conditions highlights their potential as effective bioelicitors for enhancing crop resilience and quality, particularly in water-limited environments. This mechanistic understanding not only clarifies the physiological basis for the observed improvements in guar growth and yield parameters but also suggests potential pathways for further optimizing these treatments within sustainable agricultural practices.

Our findings on the effects of MeJA and CH on guar under water stress are consistent with previous research on various crops. Studies on “Anna” apple seedlings [51], *Arachis hypogaea* L. hairy roots [52], *Phaseolus vulgaris* L. [53], and *Lupinus termis* L. [42] demonstrated the efficacy of these elicitors in mitigating water-deficit stress and enhancing the production of secondary metabolites like total phenolic content and flavonoid. Specifically, our study shows that MeJA and CH, both individually and in combination, significantly improved the content of secondary metabolites (total phenolic content, flavonoid, and anthocyanin) in guar varieties under well-watered, moderate, and severe stress conditions. This enhancement in secondary metabolites correlates with improved plant performance, likely due to stomatal closure, reduced transpiration, and increased photosynthesis under water-deficit stress [53,54,55].

Importantly, our research represents the first report of the co-administration of MeJA and CH in guar varieties to enhance phenolic content, flavonoid, and anthocyanin synthesis under water-deficit stress. The success of this innovative approach is in line with previous positive findings in other crops such as *Phaseolus vulgaris* L., where CH application reduced the effects of water-deficit stress and enhanced antioxidant activity [53], and in fodder corn, where MeJA treatment significantly increased soluble carbohydrates and mitigated the effects of drought stress [56]. Our results not only highlight the potential of combined MeJA and CH treatments to enhance guar stress resistance but also suggest a promising strategy for improving crop quality and yield under moderate water-deficit stress. The consistent positive effects observed across different stress levels underline the versatility of these elicitors and their potential applications in different agricultural scenarios, ranging from optimal to sub-optimal growing conditions.

The impact of irrigation regimes on guar gum content and quality has been a topic of debate in the literature. Our results demonstrated a decrease in gum percentage under water-deficit stress conditions, aligning with the findings, who reported that higher irrigation regimes resulted in increased gum content [18]. However, other studies suggest that gum content is a quantitative trait influenced by multiple genes and may not be significantly affected by water-deficit stress or genotype [1]. This discrepancy highlights the complex nature of gum production in guar and indicates that the response to water-deficit stress may be influenced by various factors, including genetic background and specific environmental conditions. The decrease in the gum percentage observed in our study under water-deficit stress can be attributed to the reduced photosynthetic rate, impaired nutrient uptake, and altered carbohydrate metabolism associated with such stress [54]. Although guar gum is primarily composed of polysaccharides, the synthesis and accumulation of which may be disrupted under water-deficit stress, this can occur due to reduced photosynthetic activity. Plants under moderate water-deficit stress typically increase their production of secondary metabolites as an adaptive response. In guar, this stress response mechanism initially results in increased gum production. However, when water stress exceeds the plant’s genotype-specific tolerance threshold, both photosynthetic capacity and secondary metabolism are compromised, resulting in reduced water tolerance and consequently reduced gum content [54]. This biphasic response illustrates the delicate balance between stress-induced defense mechanisms and the point at which stress becomes detrimental to plant metabolism. Similar beneficial effects have been observed in other crops, such as increased soluble carbohydrates in feed maize under drought stress with MeJA application [56] and enhanced protein and carbohydrate in *Phaseolus vulgaris* [55] and *Lupinus termis* seeds with CH treatment under water-deficit stress [42]. The efficacy of MeJA, CH, and their combination in improving guar quality traits, even under well-watered conditions, suggests their potential as elicitors beyond mere stress mitigation. Since there is a significant demand for guar plants in many different industries and drought stress, one of the effects of climate change is getting worse, it makes sense to use the treatments employed in this study to produce these guar types in the modern world.

## 5. Conclusions

This study demonstrates that while water-deficit stress negatively impacts the growth, yield, and quality traits of guar, the application of elicitors, particularly methyl jasmonate (MeJA), chitosan (CH), and their combination (MeJA + CH), can significantly mitigate this adverse effect on guar quantity and quality traits. Foliar application of MeJA at 25 µM or CH at 150 mg/L, applied three times during critical growth stages, improved morphological traits, 1000-seed weight, yield, and gum quality attributes in both varieties, under both well-watered and water-deficit stress conditions. Individual treatments of MeJA and CH exhibited more pronounced effects than their combined application in enhancing yield components. In contrast, the combination of MeJA + CH was particularly effective in increasing secondary metabolite production (total phenolic content, flavonoid, and anthocyanin) as well as quality attributes such as gum percentage and viscosity. The application of these elicitors improved gum percentage and viscosity in both RGC-986 and BR-2017 varieties under well-watered, moderate, and severe water-deficit stress conditions. This improvement can be attributed to the ability of MeJA, CH, and their combination (MeJA + CH) to promote proline and soluble sugar accumulation, increase the net photosynthetic rate and relative leaf water content, and modulate endogenous abscisic acid levels while inhibiting lipid peroxidation. These improvements are attributed to enhanced water availability and secondary metabolism under water-deficit stress. Our findings highlight the practical potential of MeJA, CH, and MeJA + CH as cost-effective strategies to optimize guar yield and quality, especially under water-deficit conditions, by harnessing the physiological and metabolic improvements induced by MeJA, CH, and MeJA + CH treatments. This research provides valuable insights for developing sustainable agronomic practices in arid and semi-arid regions where water scarcity significantly limits crop production. Future studies should investigate the long-term effects of these treatments on different guar varieties and refine application protocols for field conditions, thereby contributing to more resilient and productive guar cropping strategies.

## Figures and Tables

**Table 1:** Analysis of variance (ANOVA) indicating the effects of guar varieties (RGC-986 and BR-2017) under water-deficit stress and the different levels of methyl jasmonate (MeJA), chitosan (CH) elicitors, and their combination.

S.O.V	df	Plant Height	Pod Length	Number of Pods Plant^−1^	Number of Seeds Pod^−1^	Seed Yield	1000-Seed Weight	Biomass	Root Length	Root Dry Weight	Total Phenolic Content	Total Flavonoid	Anthocyanin Content	Gum Percentage	Viscosity
Replication	2	36.07	0.35	0.29	0.527	0.01	1.19	1.27	1.39	0.01	0.01	0.005	0.005	3.79	32,156.76
Variety (V)	1	39.27 **	2.25 **	7.56 **	3.062 **	0.39 **	9.36 **	14.18 **	1360.38**	0.02 *	17.10 **	0.057 **	0.004	238.08 **	4,964,505.84 **
Irrigation (I)	2	5334.97 **	1.89 **	5.36 **	2.194 **	0.36 **	509.90 **	18.38 **	113.67 **	0.18 **	2.93 **	0.059 **	0.043 **	93.32 **	4,692,066.29 **
Elicitors (E)	7	409.69 **	2.19 **	2.04 **	3.499 **	0.13 **	105.79 **	2.42 **	97.26 **	0.09 **	0.88 **	0.048 **	0.130 **	13.34 **	910,667.12 **
V × I	2	1956.02 **	1.70 **	2.33 **	2.333 **	0.05 **	159.48 **	2.32 *	68.10 **	0.06 **	2.93 **	0.015 **	0.004	7.82 **	701,274.87 **
V × E	7	90.62 **	0.42 **	0.37	0.872 **	0.05 **	10.10 **	1.02	8.09 **	0.02 **	0.28 **	0.005 **	0.009	4.63 **	103,574.51 **
I × E	14	124.01 **	0.45 **	0.34	0.424 **	0.02 **	9.00 **	0.86	23.34 **	0.01 **	0.19 **	0.002 *	0.006 *	3.97 **	171,432.97 **
V × I × E	14	86.83 **	0.72 **	0.81 **	0.881 **	0.04 **	9.29 **	0.71 **	22.95 **	0.02 **	0.21 **	0.002 **	0.005	2.82 **	88,528.72 **
CV (%)	-	4.75	6.28	15.94	12.458	16.36	3.35	32.06	4.39 **	14.29	9.69	10.398	8.567 **	3.29	4.11

*, ** significant at *p* ≤ 0.05 and *p* ≤ 0.01, respectively, according to Tukey’s honest significant difference (HSD) test.

**Table 2:** Mean comparison for the plant height and pod length traits of guar (RGC-986) as affected by elicitors under different irrigation regimes.

Traits	Irrigation Regimes (I)			Elicitors					
		Control	MJ1	MJ2	MJ3	CH1	CH2	CH3	MJ2 + CH2
Plant height (cm)	I1	40.5ij	40.33ij	43.00g–i	43.33g–i	41.67hi	46.67e–g	47.17d–f	45.33f–h
	I2	42.33hi	41.33i	43.67f–i	50.67d	61.33b	65.33a	55.67c	59.67b
	I3	23.67l	34.33k	40.67ij	49.33de	37.33jk	37.33jk	36.33k	35.67k
Pod length (cm)	I1	4.17fg	4.17fg	5.00bc	5.17b	5.00bc	4.5d–f	4.50d–f	5.00bc
	I2	4.00g	4.33e–g	4.50d–f	4.83b–d	4.33e–g	4.67c–e	4.33e–g	5.00bc
	I3	5.00bc	5.83a	4.83b–d	5.17b	4.50d–f	6.00a	5.17b	5.00bc

Different letters within the same column indicate significant differences between means according to Tukey’s honest significant difference (HSD) test (*p* ≤ 0.05); irrigation regimes-I1: 100% FC (field capacity), I2: 70% FC, I3: 40% FC, elicitors: MJ1: 5 μM methyl jasmonate (MJ), MJ2: 25 μM, MJ3: 50 μM, chitosan (CH), CH1: 100 mg/L, CH2: 150 mg/L, CH3: 200 mg/L.

**Table 3:** Mean comparison for the plant height and pod length traits of guar (BR-2017) as affected by elicitors under different irrigation regimes.

Traits	Irrigation Regimes (I)				Elicitors				
		Control	MJ1	MJ2	MJ3	CH1	CH2	CH3	MJ2 + CH2
Plant height (cm)	I1	42.67i	63.83bc	70.33a	66.73b	55.5e	62.5cd	59.33d	51.67f
	I2	35.17j	45.33g–i	47.33g	46.33gh	43.83hi	50.67f	63.33c	55.67e
	I3	22.33n	26.67lm	30.33k	34.00j	26.17m	27.67k–m	29.67kl	30.67k
Pod length (cm)	I1	4.67d–f	4.83c–e	5.17b–d	5.00b–e	5.00b–e	5.00b–e	7.17a	5.50b
	I2	4.17fg	4.50e–g	4.83c–e	5.00b–e	4.50e–g	5.33bc	4.83c–e	5.17b–d
	I3	5.00b–e	5.00b–e	5.00b–e	4.83c–e	5.50b	4.83c–e	5.17b–d	5.00b–e

Different letters within the same column indicate significant differences between means according to Tukey’s honest significant difference (HSD) test (*p* ≤ 0.05); irrigation regimes-I1: 100% FC (field capacity), I2: 70% FC, I3: 40% FC, elicitors: MJ1: 5 μM methyl jasmonate (MJ), MJ2: 25 μM, MJ3: 50 μM, chitosan (CH), CH1: 100 mg/L, CH2: 150 mg/L, CH3: 200 mg/L.

**Table 4:** Mean comparison for the number of pods and seed yield traits of guar (RGC-986) as affected by elicitors under different irrigation regimes.

Traits	Irrigation Regimes (I)					Elicitors			
		Control	MJ1	MJ2	MJ3	CH1	CH2	CH3	MJ2 + CH2
Number of pods plant^−1^	I1	3.00bc	2.33c	3.00bc	3.33ab	3.00bc	3.33ab	3.00bc	3.33ab
	I2	2.67bc	3.00bc	3.00bc	3.33ab	2.67bc	3.33ab	3.33ab	2.67bc
	I3	2.33c	2.67bc	4.00a	3.00bc	2.33c	2.33c	2.67bc	3.00bc
Number of seeds pod^−1^	I1	2.67ef	2.33f	3.00de	3.33cd	3.00de	3.33cd	3.00de	3.33cd
	I2	2.67ef	3.00de	3.00de	3.33cd	2.67ef	3.67bc	3.00de	3.00de
	I3	3.00de	4.00b	5.00a	4.00b	3.00de	3.33cd	3.00de	3.00de
Seed yield per plant (g)	I1	0.41d–i	0.46b–g	0.57a	0.56ab	0.52a–c	0.53a–c	0.44c–h	0.51a–d
	I2	0.33ij	0.34ij	0.45c–h	0.47b–g	0.35h–j	0.39f–j	0.36h–j	0.43c–i
	I3	0.41d–j	0.48a–f	0.50a–e	0.33ij	0.37g–j	0.31j	0.40e–j	0.45c–h

Different letters within the same column indicate significant differences between means according to Tukey’s honest significant difference (HSD) test (*p* ≤ 0.05); irrigation regimes-I1: 100% FC (field capacity), I2: 70% FC, I3: 40% FC, elicitors: MJ1: 5 μM methyl jasmonate (MJ), MJ2: 25 μM, MJ3: 50 μM, chitosan (CH), CH1: 100 mg/L, CH2: 150 mg/L, CH3: 200 mg/L.

**Table 5:** Mean comparison for the number of pods and seed yield traits of guar (BR-2017) as affected by elicitors under different irrigation regimes.

Traits	Irrigation Regimes (I)					Elicitors			
		Control	MJ1	MJ2	MJ3	CH1	CH2	CH3	MJ2 + CH2
Number of pods plant^−1^	I1	3.33c–e	4.33b	4.33b	5.33a	3.67b–d	3.33c–e	4.00bc	3.67b–d
	I2	2.67e–g	3.33c–e	4.33b	3.00d–f	3.33c–e	3.00d–f	3.33c–e	3.33c–e
	I3	2.00g	2.33fg	3.00d–f	4.00bc	2.67e–g	3.00d–f	3.00d-f	3.33c–e
Number of seeds pod^−1^	I1	3.00e–g	4.33bc	4.33bc	5.33a	3.67c–e	3.33d–f	3.00e–g	3.33d–f
	I2	2.33g	3.33d–f	4.33bc	3.00e–g	3.33d–f	3.00e–g	2.67fg	3.33d–f
	I3	2.33g	3.33d–f	4.00b–d	4.67ab	3.67c–e	3.00e–g	3.00e–g	4.00b–d
Seed yield per plant (g)	I1	0.82bc	0.83ab	0.97a	0.69b–d	0.45f–k	0.55d–g	0.67c–e	0.29l
	I2	0.54e–h	0.73bc	0.46f–k	0.55d–f	0.35j–l	0.48f–j	0.55d–g	0.27l
	I3	0.32kl	0.40g–l	0.82bc	0.37i–l	0.49f–j	0.40h–l	0.52f–i	0.32kl

Different letters within the same column indicate significant differences between means according to Tukey’s honest significant difference (HSD) test (*p* ≤ 0.05); irrigation regimes-I1: 100% FC (field capacity), I2: 70% FC, I3: 40% FC, elicitors: MJ1: 5 μM methyl jasmonate (MJ), MJ2: 25 μM, MJ3: 50 μM, chitosan (CH), CH1: 100 mg/L, CH2: 150 mg/L, CH3: 200 mg/L.

## Data Availability

The data presented in this study are available upon request from the corresponding author.
